# Factors increasing the risk for psychosocial stress among Korean adults living in rural areas: using generalized estimating equations and mixed models

**DOI:** 10.1186/s40557-017-0209-5

**Published:** 2017-10-31

**Authors:** Ju-Hyun Nam, Myeong-Seob Lim, Hyun-Kyeong Choi, Jae-Yeop Kim, Sung-Kyeong Kim, Sung-Soo Oh, Sang-Baek Koh, Hee-Tae Kang

**Affiliations:** Department of Occupational and Environmental Medicine, Wonju Severance Christian Hospital, Wonju College of Medicine, Yonsei University, Wonju, Korea

**Keywords:** Sleep duration, Exercise, Sedentary time, Stress, Population-based study, ARIRANG cohort

## Abstract

**Background:**

This study was conducted to analyze the distribution of the psychosocial well-being index among adults living in two rural communities in Korea and to examine its correlation with lifestyle variables such as sleep duration, regular exercise, and sedentary time.

**Methods:**

Using the cohort data of the Atherosclerosis Risk of a Rural Area Korean General Population, this study examined 3631 participants living in Wonju and Pyeongchang in Gangwon Province; their preliminary data were established from 2005 to 2007 while their follow-up data were collected 3 years later. This study investigated demographic characteristics, lifestyle habits, disease history, Psychosocial Well-being Index-Short Form (PWI-SF) scores, sleep duration, regular exercise, and sedentary time during work. Using repeated measures ANOVA, this study examined how the variables and PWI-SF scores changed over the course of 3 years and identified the correlation between them based on mixed model analysis. Afterwards, using the generalized estimation equation, this study identified each variable’s risk towards the PWI-SF high-risk group and performed a stratified analysis by occupation after dividing the participants into farmers and non-farmers.

**Results:**

The PWI-SF high-risk group was found to be 18.9% of the participants from preliminary data and 15.5% from follow-up data. The odds ratio towards the PWI-SF high-risk group was 1.503 (95% CI 1.241–1.821) in the short sleep duration group and 1.327 (95% CI 1.136–1.550) in the non-regular exercise group. A stratified analysis by occupation showed that middle and long sedentary time in the white-collar group increased the risk toward the PWI-SF high-risk group.

**Conclusions:**

Short sleep duration, no regular exercise, and long sedentary time in the white-collar group were identified as risk factors toward the PWI-SF high-risk group in the rural communities, and policy interventions are needed to address this issue.

## Background

Stress refers to tension or anxiety that arises in response to new needs or changes in the external environment, and it can be detrimental to quality of life when it is severe. In modern times, stress reduction has become a critical factor in improving the health of people, and various approaches have been attempted to relieve it [1–3].

The notion that excessive stress results in adverse health consequences has been extensively documented. Several studies have revealed that elevated stress not only increases the incidence of obesity, cardiovascular disease, and cerebrovascular disease [4–6], but it also is related to psychosocial problems, such as insomnia and depression [7, 8]. In addition, stress also stimulates individuals to adopt poor health-related lifestyle habits, such as smoking and drinking alcohol, and in turn, various health-related lifestyle habits, such as reduced sleep duration, lack of exercise, and sedentary behaviors that may alter levels of stress [9–13]. Among these factors, exercise, time spent sitting, and sleep account for the majority of a person’s day and can be actively controlled by the person. These factors have attracted much research attention to the topic with the goal of improving health and reducing the risk of preventable diseases. Most existing studies, however, are cross-sectional studies that examine data at a specific point in time, and this type of analysis has difficulty in identifying how the level of stress changes over time in accordance with lifestyle habits. In this regard, this study aims to examine how lifestyle habits and the level of stress changed over time by using preliminary data, which were collected over the course of 3 years, and follow-up data, which were collected 3 years later.

Using the generalized estimating equation (GEE), this study was performed to identify a correlation between the psychosocial well-being index and lifestyle variables (sleep duration, regular exercise, and sedentary time) among adults in 2 rural areas. In addition, conducting a stratified analysis by occupation on farmers and non-farmers, this study intended to identify additional features of the rural areas.

## Methods

### Design and participants

This study analyzed cohort data that were collected as part of the Atherosclerosis Risk in Rural Areas in the Korean General Population (ARIRANG) survey. The participants consisted of adults living in the rural areas of Wonju and Pyeongchang in Gangwon Province, South Korea. The ARIRANG cohort data were collected from 5178 adults, aged 40 and older (2127 men and 3051 women), from November 2005 to January 2008 and the follow-up data were collected from 3862 adults from April 2008 to January 2011. Thus, the data comprise two panels with a mean follow-up period of 2.8 years. Among the 3862 adults who completed the follow-up survey, 231 were excluded because they omitted one or more responses to items on the Psychosocial Well-being Index-Short Form (PWI-SF), resulting in 3631 adults in the final analysis.

### Psychosocial Well-being Index-Short Form (PWI-SF)

Stress, the dependent variable of this study, was measured using the PWI-SF. This 18-item instrument was constructed based on Goldberg’s General Health Questionnaire (GHQ-60) and its items and scales were revised over two trials [14]. The items are rated on a 4-point (0–3) Likert scale, ranging from “very true,” “mostly true,” “slightly true,” and “not at all true”. Ratings for each item (0–3) are summed for a total score ranging from 0 to 54 points, with a higher score indicating greater psychosocial stress. The total score is classified as “healthy” (< 8 points), “potential stress” (9–26 points), and “high risk” (> 27 points) [15]. However, in the present study, a dichotomous classification was adopted, with scores in the “healthy” and “potential stress” categories, classified as “low risk” and the other scores as “high risk”.

### Lifestyle variables

The main explanatory variables of this study consisted of three lifestyle measures: sleep duration, exercise, and time spent sitting or sedentary time at work. These variables were measured during the preliminary and follow-up surveys.

Sleep duration was measured using the following item: “What has been your average daily sleep duration (including naps) for the past year?” The response options were: “less than six hours,” “six to nine hours,” and “more than 10 h.” Based on previous studies on sleep duration [16, 17], the adults with less than 6 h of sleep per day, were classified as the short-sleep group, adults with 6 to 9 h per day, as the moderate-sleep group, and those with more than 10 h of sleep per day, as long-sleep group.

Regular exercise was measured using the following item: “Do you regularly perform exercise that is intense enough to make you sweat?” The response options were “I do not” and “I do.” People who marked “I do” were as assigned to the regular-exercise group and those who marked “I do not” were assigned to the no-regular-exercise group.

Sedentary behaviors were examined using the following question: “How many hours per day have you spent sitting during work over the past 1 year?” The workers were divided into terciles: the *high frequency* sedentary behavior group with 5 h or more per day, the *medium frequency* group with 1 to 5 h, and the *low frequency* group with less than 1 h. Preliminary data used only the question above to measure sedentary behaviors while follow-up data had 5 sedentary behavior questions in total including the aforementioned one. To obtain result values using the GEE, however, this study used the question included in both panel data. As the question asks about sedentary time spent specifically during work, this study excluded housewives and unemployed individuals and included only those participants who had occupations in its analysis based on sedentary behaviors. Occupations of the respondents in the questionnaire were coded in accordance with the *Korean Standard Classification of Occupations 2007* [18].

### Potential confounding variables

We used several potential confounding variables and included factors that have been reported to be associated with stress in multiple studies [6, 7, 11]. The participants’ characteristics were classified as demographic, lifestyle, and medical, all of which were investigated in both the preliminary and follow-up surveys. The specific variables are shown below. Highest level of education and monthly income were not investigated in the follow-up survey; therefore, the follow-up data for the two variables were replaced with the results from the preliminary survey.

- Demographic variables: Sex (male/female), age (40–49 years, 50–59 years, ≥ 60 years), highest level of education (lower than elementary, middle school, high school, or two-year college or higher), monthly income (<1 million won, 1–2 million won, 2–3 million won or >3 million won), marital status (spouse (married or living together) or no spouse (divorced, separated, widowed or single)
- Occupation: job class(farmers, livestock farmers/ blue-collar^1^/white-collar^2^/housewives or unemployed), the average number of working hours per day (8 to 10 h, less than 8 h, and 10 h or more)
- Lifestyle variables: smoking (non-smoker, past smoker, or current smoker), drinking alcohol (yes/no)
- Medical variables: BMI (kg/m^2^), chronic metabolic disease (hypertension, diabetes, dyslipidemia, coronary artery disease (ischemic heart disease, myocardial infarction), cerebrovascular disease (stroke, transient ischemic attack)).

### Statistical analysis

The generalized estimation equation (GEE) is used to estimate a causal model between panels or for an entire panel of data, and is a useful technique when dealing with repeated measures or time-series data. Particularly, the GEE can be used to calculate asymptotic estimates based on quasi-likelihoods even in cases in which the correlations between the explanatory and dependent variables are unknown or there are missing partial correlations for the explanatory variables [19].

To begin, we used the chi-squared test and paired t-test and compared the variables to see if there had been any change between preliminary data and follow-up data. Then, using repeated measures analysis of variance (RM-ANOVA), this study analyzed how the variable groups changed over 3 years with all categorical variables went through dummy coding. RM-ANOVA followed by post-hoc Bonferroni correction was performed for all variables. Next, using the GEE, this study calculated all variables’ odds ratio (OR) and 95% confidence interval (CI) of the risk towards the PWI-SF high-risk group. Also, a mixed model analysis was performed. For each variable, ß and 95% confidence interval (CI) were presented. Additionally, this study performed a stratified analysis by occupation. This study also conducted the GEE analysis on farmers, blue-collar workers, and white-collar workers for their lifestyle variables and calculated each of the variables’ OR and 95% CI of the risk towards the PWI-SF high-risk group.

To indicate statistical significance, *p* < 0.05 was applied. All data analyses were performed using the SPSS program (SPSS [IBM Corp. Released 2013. IBM SPSS Statistics for Windows, Version 22.0. Armonk, NY: IBM Corp.]).

## Results

### Demographic characteristics

Table 1 shows the general characteristics of the participants in the preliminary and follow-up surveys. Male and female participants accounted for 41.8% and 58.2%, respectively, of the total sample. Participants’ age in the preliminary survey ranged from 40 to 73 years, with a mean age of 55.32 years. A total of 18.9% of the participants in the preliminary survey and 15.5% in the follow-up survey were at high risk for psychosocial stress. The total PWI score was 18.74 points in the preliminary survey and 16.70 points in the follow-up survey, showing a significant decline over time. The sleep duration of the vast majority of the participants was in the moderate range (84.9%), followed by those in the short-sleep (10.9%), and long-sleep (4.2%) groups in the preliminary survey. In the follow-up survey, the long-sleep group decreased to 2.0%, whereas the moderate-sleep group increased to 87.1%. The regular-exercise group accounted for 31.6% of the sample, indicating that a higher proportion of participants did not exercise regularly, and the proportion of adults who did exercise regularly, significantly increased in the follow-up survey (34.6%). By occupation, farmers accounted for the largest percentage with 43.0% in preliminary data and 41.8% in follow-up data followed by blue-collar and white-collar workers. For sedentary time during work, the number of workers with less than 1 h was the highest among the participants excluding housewives and unemployed individuals, and the percentage of those with 5 h or more increased slightly in follow-up data. There were significant differences between preliminary and follow-up data for average number of working hours per day, smoking, and chronic metabolic disease.

**Table 1:** General characteristics of all study participants (*n* = 3631)

Variables	Mean ± SD or percentage(%)				*p value*
	Preliminary survey (2005–2007)		Follow-up surveys (2008–2010)		
	n	%	n	%	
Dependent variable					
PWI total score	18.74 ± 8.94		16.70 ± 9.59		*<0.001* ^†^
PWI high risk group	18.9%		15.5%		*<0.001* ^†^
Lifestyle variables					
Sleep duration					0.035*
6 to 9 h	3081	84.9	3162	87.1	
Less than 6 h	395	10.9	396	10.9	
More than 10 h	115	4.3	73	2.0	
Regular exercise					<0.001*
Yes	1147	31.6	1256	34.6	
No	2484	68.4	2375	65.4	
Smoking					<0.001*
Non-smoker	2211	60.9	2128	58.6	
Past smoker	821	22.6	947	26.1	
Current smoker	599	16.4	556	15.3	
Drinking alcohol					0.103*
No	2102	57.9	2041	56.2	
Yes	1529	42.1	1590	43.8	
Medical variables					
BMI (kg/m^2^)	24.65 ± 3.13		24.30 ± 3.19		*0.281* ^†^
Chronic metabolic disease (number)					<0.001*
0	2622	72.2	2394	66.9	
1	802	22.1	940	25.9	
2	174	4.8	243	6.7	
3 or more	33	0.9	54	1.5	
Occupational variables					
Occupation class					0.013*
Farmers, livestock farmers	1563	43.0	1516	41.8	
Blue-collar	609	16.8	587	16.2	
White-collar	397	10.9	397	10.9	
Housewives or unemployed	1062	29.3	1131	31.1	
Working hours per day					0.021*
8 to 10 h	1030	28.4	1077	29.6	
Less than 8 h	529	14.6	555	15.3	
More than 10 h	1150	31.6	1098	30.3	
None	922	25.4	901	24.8	
Sedentary time at work^a^					<0.001*
Less than 1 h per day	1018	39.8	1021	39.3	
1 to 5 h per day	939	36.6	921	35.4	
More than 5 h per day	603	23.6	658	25.3	
Demographic variables					
Sex					
Male	1518	41.8	1518	41.8	
Female	2113	58.2	2113	58.2	
Age	55.32 ± 8.24		58.15 ± 8.19		*0.011* ^†^
40–49 years	861	23.7	719	19.8	0.003*
50–59 years	1336	36.8	1314	36.2	
≥ 60 years	1434	39.5	1598	44.0	
Highest level of education^b^					
Lower than elementary	1808	49.8			
Middle school	650	17.9			
High school	792	21.8			
Two-year college or higher	381	10.6			
Monthly income^b^					
< 1 million won	1565	43.1			
1–2 million won	1031	28.4			
2–3 million won	479	13.2			
> 3 million won	556	15.4			
Marital status					0.089*
Spouse	3260	89.8	3210	88.4	
No spouse	371	10.2	421	11.6	

*by χ2-test

^†^by paired T-test

^a^Housewives and unemployed were excluded

^b^Highest level of education and monthly income were not investigated in the follow-up survey

Each group’s changes and differences over 3 years in the PWI-SF score were analyzed using RM-ANOVA, and the results are provided in Table 2. It was found that the PWI-SF score of all the groups significantly decreased over the 3 years (*p* < 0.001, data not shown). The variables that showed significant changes in the PWI-SF score over time included sleep duration, regular exercise, smoking history, number of chronic diseases, occupation, average number of working hours per day, sex, highest level of education attained, average monthly income, and marital status.

**Table 2:** RM-ANOVA results of all categorical variables

Variables	Group	Preliminary survey	Follow-up surveys	% change	*p value*
		M ± SD	M ± SD		
Overall		18.74 ± 8.94	16.70 ± 9.59	−10.9%	
Sleep duration	6 to 9 h	18.38 ± 8.67	16.42 ± 9.44	−10.7%	<0.001*
	Less than 6 h	21.57 ± 9.97	18.66 ± 10.33	−13.5%	
	More than 10 h	18.67 ± 9.98	17.15 ± 10.10	−8.1%	
Exercise	Yes	17.05 ± 8.19	15.13 ± 8.58	−11.3%	<0.001*
	No	19.51 ± 9.16	17.42 ± 8.58	−10.7%	
Smoking	Non-smoker	19.38 ± 9.13	17.19 ± 9.82	−11.3%	<0.001*
	Past smoker	16.45 ± 7.93	14.61 ± 8.40	−11.2%	
	Current smoker	17.76 ± 8.44	16.14 ± 9.22	−9.1%	
Drinking alcohol	No	18.66 ± 8.83	16.69 ± 9.62	−10.7%	*0.927*
	Yes	18.90 ± 9.09	16.65 ± 9.56	−10.9%	
Chronic metabolic disease (number)	0	18.31 ± 8.75	16.20 ± 9.32	−11.5%	<0.001*
	1	19.37 ± 9.35	17.51 ± 9.92	−9.6%	
	2	20.15 ± 8.84	17.96 ± 10.28	−10.9%	
	3	21.96 ± 10.10	21.48 ± 11.12	−2.2%	
Occupational class	Farmers, livestock farmers	18.99 ± 9.47	17.01 ± 10.04	−10.4%	<0.001*
	Blue-collar	18.34 ± 8.91	16.62 ± 8.65	−10.6%	
	White-collar	17.70 ± 9.00	15.75 ± 9.01	−11.0%	
	Housewives or unemployed	19.38 ± 8.72	16.98 ± 9.55	−13.4%	
Working hours per day	8 to 10 h	18.00 ± 8.73	16.33 ± 9.44	−9.3%	0.039*
	Less than 8 h	18.08 ± 8.80	17.05 ± 9.55	−5.7%	
	More than 10 h	19.21 ± 9.33	17.02 ± 9.89	−11.4%	
	None	19.33 ± 8.67	16.51 ± 9.39	−14.6%	
Sedentary time on work^a^ (per day)	Less than 1 h	19.14 ± 9.10	16.52 ± 9.71	−13.7%	*0.155*
	1 to 5 h	18.00 ± 8.90	16.61 ± 9.58	−7.7%	
	More than 5 h	17.58 ± 8.67	15.98 ± 9.42	−9.1%	
Sex	Male	16.53 ± 8.29	14.71 ± 9.03	−11.0%	<0.001*
	Female	20.32 ± 9.05	18.13 ± 9.73	−10.8%	
Age	40–49 years	18.33 ± 8.27	16.20 ± 8.28	−10.6%	*0.099*
	50–59 years	18.74 ± 8.82	16.98 ± 9.35	−9.4%	
	≥ 60 years	19.07 ± 9.55	16.81 ± 10.75	−11.9%	
Highest level of education	Lower than elementary	20.21 ± 9.64	17.88 ± 10.44	−11.5%	<0.001*
	Middle school	18.05 ± 8.08	16.72 ± 9.25	−7.4%	
	High school	17.34 ± 7.74	15.04 ± 8.13	−13.3%	
	Two-year college or higher	15.65 ± 7.73	14.54 ± 7.74	−7.1%	
Monthly income	< 1 million won	20.13 ± 9.61	18.00 ± 10.54	−10.5%	<0.001*
	1–2 million won	18.61 ± 8.37	16.32 ± 8.76	−12.2%	
	2–3 million won	16.85 ± 7.92	14.78 ± 8.25	−12.3%	
	> 3 million won	16.10 ± 7.11	14.57 ± 7.87	−9.5%	
Marital status	Spouse	18.41 ± 8.70	16.53 ± 9.46	−10.2%	0.004*
	No spouse	21.51 ± 10.39	18.17 ± 10.63	−15.5%	

*M* mean, *SD* standard deviation

*Significant at the 0.05 level

^a^Housewives and unemployed were excluded

### Effects of sleep duration, exercise, and sedentary time on the likelihood of being at high risk for psychosocial stress

Table 3 shows each variable’s OR and 95% CI of the risk towards the PWI-SF high-risk group from the GEE analysis on all participants. Significant outcomes were identified across all participants in the rural areas depending on sleep duration and regular exercise. The less-than-6-h sleep duration group showed a risk factor towards to the PWI-SF high-risk group 1.503 times (95% CI 1.241–1.821) higher than the medium sleep duration group while the non-regular exercise group had a risk factor 1.327 times (95% CI 1.136–1.550) higher than their counterpart group. In other lifestyle habits, smokers showed an OR 1.329 times (95% CI 1.069–1.652) higher than non-smokers, but there was no significant difference when it came to drinking history. With regard to the occupation variable, blue-collar workers were 1.165 times (95% CI 1.022–1.465) more likely to enter the PWI-SF high-risk group than farmers, but white-collar workers did not show statistically significant differences (OR 1.031, 95% CI 0.817–1.301). The group with 10 working hours or more showed a higher risk (OR 1.181, 95% CI 1.006–1.417) than the group with 8 to 10 working hours. In addition, being female, having less than KRW 2 million in monthly income, having middle school graduation or lower education level, and having at least 1 chronic disease worked as risk factors towards the PWI-SF high-risk group.

**Table 3:** GEE estimations and mixed model analysis to PWI-SF high risk group of each variables

	GEE		Mixed model	
	OR	95% CI	ß	95% CI
Lifestyle variables				
Sleep duration^a^				
6 to 9 h	1.000			
Less than 6 h	1.503	1.241–1.821	2.393	1.947–2.839
More than 10 h	1.026	0.845–1.245	0.241	−0.471-0.951
Regular exercise^a^				
Yes	1.000			
No	1.327	1.136–1.550	1.686	1.358–2.014
Smoking^a^				
Non-smoker	1.000			
Past smoker	1.195	0.928–1.537	0.540	−0.187−1.273
Current smoker	1.329	1.069–1.652	1.545	1.040–2.050
Drinking alcohol^a^				
No	1.000			
Yes	0.980	0.842–1.140	0.179	−0.115−0.473
Medical variables				
BMI (kg/m^2^)	1.021	1.000–1.043	−0.096	−0.143−(−0.049)
Chronic metabolic disease (number)^a^				
0	1.000			
1	1.196	1.007–1.422	1.252	0.897–1.611
2	1.511	1.177–1.940	2.386	1.822–2.950
3 or more	2.452	1.591–3.779	4.645	3.463–5.827
Occupational variables				
Occupation class^a^				
Farmers, livestock farmers	1.000			
Blue-collar	1.165	1.022–1.465	0.301	0.112–0.490
White-collar	1.031	0.817–1.301	−0.512	−1.152−0.128
Housewives or unemployed	0.846	0.707–1.012	−0.336	−0.887−0.215
Working hours per day^a^				
8 to 10 h	1.000			
Less than 8 h	0.986	0.754–1.290	−0.662	−0.998−(−0.286)
More than 10 h	1.181	1.006–1.417	0.255	0.054–0.451
None	0.942	0.705–1.257	0.559	0.050–1.068
Demographic variables				
Sex^a^				
Male	1.000			
Female	2.341	1.876–2.920	3.816	3.379–4.253
Age^a^				
40–49 years	1.000			
50–59 years	1.134	0.918–1.399	−0.115	−0.093−0.323
≥ 60 years	1.105	0.887–1.378	0.094	−0.061−0.249
Highest level of education^a^				
Two-year college or higher	1.000			
High school	1.092	0.804–1.766	0.769	0.349–1.189
Middle school	1.603	1.075–2.389	1.141	0.708–1.574
Lower than elementary	1.909	1.291–2.822	1.477	0.893–2.062
Monthly income^a^				
> 3 million won	1.000			
2–3 million won	1.078	0.720–1.616	0.396	−0.119−0.911
1–2 million won	1.662	1.185–2.331	0.988	0.589–1.387
< 1 million won	2.072	1.464–2.933	1.587	1.061–2.113
Marital status^a^				
Spouse	1.000			
No spouse	1.190	0.947–1.496	1.529	1.081–2.017

^a^Dummy-coded values were used for mixed model analysis

*SE* standard errors

The groups that showed statistically significant correlations in a mixed model analysis had results almost the same as those found significant in the GEE analysis. Some education level and marital status groups that were not significant in the GEE analysis, however, produced significant correlations in the mixed model.

The results of a stratified analysis by occupation are listed in Table 4. Among farmers, short sleep duration increased the risk towards the PWI-SF high-risk group (OR 1.700, 95% CI 1.288–2.242), but it did not show any statistical significance among blue-collar and white-collar workers. For regular exercise, a consistent trend was observed overall, and no regular exercise increased the risk towards the PWI-SF high–risk group 1.171 times (95% CI 1.025–1.443) higher among farmers, 1.245 times (95% CI 1.091–1.612) higher among blue-collar workers, and 1.519 times (95% CI 0.926–2.680) higher among white-collar workers. When it came to sedentary time during work, differences were distinctive between occupation groups. Among white-collar workers, the 1-to-5-h sedentary time group (OR 1.145, 95% CI 1.012–1.357) and the 5-h-or-more group (OR 1.584, 95% CI 1.111–2.590) showed a significantly increased risk towards the PWI-SF high-risk group compared to the less-than-1-h group, and both farmers and blue-collar workers showed ORs of less than 1. Among blue-collar workers, the 5-h-or-more sedentary time group showed a significantly reduced risk towards the PWI-SF high-risk group.

**Table 4:** GEE estimations to PWI-SF high risk group of job-specific stratification analyses

	Farmers, livestock farmers	Blue-collar^b^	White-collar^c^
	OR	OR	OR
	95% CI	95% CI	95% CI
Sleep duration			
6 to 9 h	*ref.*	*ref.*	*ref.*
Less than 6 h	1.700^a^	1.043	1.227
	1.288–2.242	0.723–1.577	0.890–1.805
More than 10 h	1.046	0.947	1.249
	0.789–1.387	0.599–1.498	0.698–2.291
Regular exercise			
Yes	*ref.*	*ref.*	*ref.*
No	1.171^a^	1.245^a^	1.519
	1.025–1.443	1.091–1.612	0.926–2.680
Sedentary time at work			
Less than 1 h	*ref.*	*ref.*	*ref.*
1 to 5 h	0.922	0.832	1.145^a^
	0.745–1.140	0.683–1.094	1.012–1.357
More than 5 h	0.925	0.781^a^	1.584^a^
	0.712–1.202	0.588–0.965	1.111–2.590

All groups are modified by sex, monthly income, education level, marital status, smoking, chronic disease, working hours per day

^a^Significant at the 0.05 level

Blue-collar^b^: craft workers, machine operating and assembling workers, elementary workers, forestry and fishery workers and all manual service workers

White-collar^c^: managers, professionals, clerks, sales workers and all non-manual service workers

## Discussion

This study analyzed the likelihood of specific variables of increasing the risk for psychosocial stress among adults in Korean rural areas using GEEs. We found that lack of sleep and lack of regular exercise were significant risk factors for placing Korean adults in rural areas at greater risk for psychosocial stress over time. These two risk factors were better predictors than were the other lifestyle habits, such as smoking, and they were as meaningful as other variables associated with health risks, such as income level, education level, and chronic diseases.

This study found that a short sleep duration of less than 6 h increased the risk towards the PWI-SF high-risk group by 1.49 times, which was consistent with the findings of various previous studies. Kessler et al. showed that the percentage of those who showed 16 points or less in the Kessler Psychological Distress Scale was higher in the 7-to-8-h sleep duration group than in the short sleep duration group [20]. In addition, Magee et al. stated that short sleep duration caused fatigue and mood and cognition disturbances and consequently led to poor self-rated health and quality of life [21]. A study using the Korean National Health and Nutritional Examination Survey also reported that having 5 sleeping hours or less increased the risk towards the stress high-risk group by 1.9 times, and this was 1.8 times more likely to lead to suicide ideation [22].

Though not yet clarified, there are several hypotheses that short sleep causes high stress, such as the influence of prefrontal cortex (PFC) and the control of the serotonergic system. PFC is associated with both arousal and mood control, and PFC is most active when our body is active, and PFC is the least active when taking sleep [23]. In addition, PFC is connected to amygdala and hypothalamus and controls mood, and sleep deprivation is consequently considered to weaken this association and to induce stress and depression [24]. Serotonin is a neurotransmitter that decreases secretion during sleep, which is known to be associated with mood disorders and depression [25]. Recent animal studies have shown a decreased response due to desensitization of serotonin receptors in sleep deprivations for several days [26].

Some of the latest studies on sleep duration demonstrate that not only short but also long sleep duration has correlations with negative mental health. Charles et al. showed that long sleep duration was correlated with a high score on the Perceived Stress Scale among police officers while Magee et al. stated that the long sleep duration group showed poorer self-rated health [21, 27]. A study conducted in Korea reported that the group with 9 sleeping hours or more was more likely to develop anxiety disorders and rely on alcohol [28]. However, this study did not produce significant correlations with the long sleep duration group in its major analysis or stratified analysis. Given the findings of other studies that there was no or limited correlations between long sleep duration and stress [22, 29], this issue remains debatable.

In addition, this study’s findings showed that no regular exercise increased the risk towards the PWI-SF high-risk group by 1.32 times. There are many studies that show correlations between physical exercise and stress. Hannan et al. proved that physical inactivity was correlated with a high level of stress [30] while Vankim et al. showed in their study on college students that high-intensity exercise was less likely to be correlated with poor mental health and perceived stress [31]. Another study reported that exercise had a positive effect on physical and emotional quality of life [10]. Furthermore, another study demonstrated that even low-intensity exercise, which does not satisfy needs required to build muscles or improve cardiovascular functions, helped to improve cognitive and performance abilities and make the brain healthier [32]. Given that this study did not examine the intensity of exercise, and many people would have not regarded low-intensity exercise (leisurely stroll and walk home and to work) as exercise when responding to the exercise question, there is a possibility that the risk towards the PWI-SF high-risk group posed by the absence of exercise would have been underestimated in this study. In this regard, an additional study conducted with a more structured questionnaire is needed to address this concern.

Exercise has been documented in several animal and clinical trials to relieve stress by simulating the release of various neurotransmitters, interleukin-6, and TNF-a [33]. In another study, exercise reduced depression and anxiety by actively stimulating the secretion of metabolites and neurotransmitters, including atrial natriuretic peptide, amine metabolite, and serotonin [1, 33, 34].

Sedentary time and regular exercise are considered correlated, and a study observed that those exercising less were more likely to have longer sedentary time habitually and vice versa [35]. Another study analyzed various physical activity times, sedentary time and sleep duration in 24 h as a single pattern, and quality of life differences between each of the subgroups; significant differences were observed [36]. This study, however, was not able to measure the entire sedentary time during the day including outside of work, and it was therefore difficult to confirm a correlation between sedentary time and exercise. It seems that a follow-up study must examine the entire sedentary time using other ARIRANG cohort data published later.

Most studies on sedentary time so far analyzed either all sedentary behaviors or breaking sedentary behaviors; thus, it seems not very easy to directly compare them with this study, which limits its scope to sedentary time during work. Many studies showed consistent findings that an increase in sedentary time or behavior has a correlation with negative mental health [37–40]. Recently, Lee et al. revealed that longer total working hours among Korean white-collar workers led to an increased risk towards the PWI-SF high-risk group [41]; even though this study believes that there is a correlation between total working hours and sedentary working hours due to the nature of the white-collar occupation, we did not directly address the correlation herein. In the future, it is necessary to conduct studies that focus on sedentary working hours, and this study could serve as a starting point for those studies.

For farmers and blue-collar workers, both groups with middle and long sedentary time during work showed ORs of less than 1. By contrast, the 5-h-or-more sedentary time group among blue-collar workers showed a completely opposite correlation, and this could be explained to some extent by the following: Mäkinen et al. found that blue-collar workers were more inactive during leisure time due to their physical load during work than white-collar workers, and this tendency strengthened when there was more physically strenuous work or they worked for 5 h or more standing or walking [42]. Inactive leisure time spent by blue-collar workers creates a negative effect on their mental health [36], and blue-collar workers have longer sedentary time during work when they have less physically strenuous work or stand and walk less; in the end, longer sedentary time among blue-collar workers could contribute to reducing their negative mental health.

This study has some limitations. First, this study’s major analysis was conducted using the GEE, and this method is based on a cross-sectional analysis and has a limitation in deriving a causal relationship. Even though this study used 2 different time periods from preliminary and follow-up data (tracked for 3 years), time lag was not used in the analysis. Our data is cohort data with a three-year interval and could be analyzed using method directly showing the change of variables like logistic regression analysis. However, some of our data, especially occupational variables and demographic variables, were missing, so 1173 participants out of 3862 who completed follow up had a partial data missing. This was a large proportion, accounting for 30% of the total, and there was a lot of concern about the analysis that excluded these personnel. There was also the opinion that 3 years might not be enough time to affect the lifestyle of PWI-SF. Finally, we selected GEE, which can include analysis of missing partial data [19], and excluded only 231 missing lifestyle variables (sleep duration, regular exercise, sedentary time) and PWI-SF variables. But it is true this study still has the limitation of cross-sectional study which cannot confirm causation. Second, data on lifestyle variables were collected using self-report questions. In a previous survey of exercise using a self-report questionnaire, the respondents’ tended to over-report their exercise habits and under-report their sedentary behaviors in an attempt to be viewed more favorably by the researchers [43]. Thus, in this study, the presence of social-desirability bias is possible, and if present, would be indicated by participants’ over- and under-estimations of exercise and sedentary behaviors, respectively. Third, our measure of sedentary behavior consisted of only one item. The question about sedentary behavior during work was asked only in the preliminary survey, which limited the stratification analyses of sedentary behaviors. However, the third and fourth panel surveys are underway for the cohort used in this study, which should provide data for more diverse analyses on sedentary behaviors in future studies. And some items of the questionnaires were omitted because they did not perform as expected. Changes in demographic characteristics (i.e., monthly income, highest level of education) were not assessed in the follow-up survey, and the amount of alcohol consumption vs. whether one drinks or not was measured in only some of the panels, thereby enabling the analysis of drinking status only. Finally, it is difficult to generalize the findings of this study, as the data were collected from a population from a specific region of one country.

Despite these limitations, a strength of this study is that it is the first study—as far as we know—that investigates time series data on stress risk factors in 2 rural communities in Korea using the GEE. By analyzing preliminary and follow-up data, this study showed not only how the level of stress changed over time but also temporal changes of various lifestyle variables. By including as many compounding variables as possible (sex, age, income, education level, marital status, smoking history, drinking history, BMI, chronic disease history, working hours, and occupation history), this study raised the persuasiveness of the findings. In addition, using a stratified analysis in the rural communities, this study found that each occupation group perceived stress differently. It also demonstrated that various lifestyle factors resulted in different levels of risk towards the PWI-SF high-risk group.

## Conclusion

This study identified that various lifestyle factors could affect the stress of people living in 2 rural communities. It also found that short sleep duration, absence of exercise, and the white-collar group’s long sedentary time acted as key factors towards the PWI-SF high-risk group. In Korea, there have been many health campaigns for quitting smoking and reducing alcohol consumption, but there has been relatively less interest in exercise or sleep; there is still some perception that working hard while cutting back on sleep is regarded as a virtue. As demonstrated in this study, however, the absence of regular exercise and short sleep duration clearly had an adverse effect on the increase of stress among all local residents in the rural communities; by resolving this, it would be possible to make their lives healthier. It is necessary to perform an additional study to analyze total sedentary hours and stress using these ARIRANG cohort data and to identify a causal relationship between them using longer-term follow-up data.

## Abbreviations

ARIRANG: Atherosclerosis Risk in Rural Areas in the Korean General Population survey
BMI: Body Mass Index
CI: Confidence interval
GEE: Generalized estimation equation
OR: Odds ratio
PWI: Psychosocial well-being index
PWI-SF: Psychosocial Well-being Index-Short Form
RM-ANOVA: Repeated Measures ANalysis Of VAriance

## Footnotes

1 Craft workers, machine operating and assembling workers, elementary workers, forestry and fishery workers, and all manual service workers

2 Managers, professionals, clerks, sales workers, and all non-manual service workers
